# Fine-Mapping, Gene Expression and Splicing Analysis of the Disease Associated *LRRK2* Locus

**DOI:** 10.1371/journal.pone.0070724

**Published:** 2013-08-13

**Authors:** Daniah Trabzuni, Mina Ryten, Warren Emmett, Adaikalavan Ramasamy, Karl J. Lackner, Tanja Zeller, Robert Walker, Colin Smith, Patrick A. Lewis, Adamantios Mamais, Rohan de Silva, Jana Vandrovcova, International Parkinson Disease Genomics Consortium (IPDGC), Dena Hernandez, Michael A. Nalls, Manu Sharma, Sophie Garnier, Suzanne Lesage, Javier Simon-Sanchez, Thomas Gasser, Peter Heutink, Alexis Brice, Andrew Singleton, Huaibin Cai, Eric Schadt, Nicholas W. Wood, Rina Bandopadhyay, Michael E. Weale, John Hardy, Vincent Plagnol

**Affiliations:** 1 Department of Molecular Neuroscience, UCL Institute of Neurology, Queen Square, London, United Kingdom; 2 Department of Genetics, King Faisal Specialist Hospital and Research Centre, Riyadh, Saudi Arabia; 3 University College London Genetics Institute, University College London, London, United Kingdom; 4 Department of Medical and Molecular Genetics, King's College London, Guy's Hospital, London, United Kingdom; 5 Institute of Clinical Chemistry and Laboratory Medicine, University Medical Centre Mainz, Mainz, Germany; 6 University Heart Center Hamburg, Clinic for General and Interventional Cardiology, Hamburg, Germany; 7 MRC Sudden Death Brain Bank Project, University of Edinburgh, Department of Neuropathology, Edinburgh, Scotland, United Kingdom; 8 School of Pharmacy, University of Reading, Whiteknights, Reading, United Kingdom; 9 Reta Lila Weston Institute of Neurological Studies, London, United Kingdom; 10 Laboratory of Neurogenetics, National Institute on Aging, National Institutes of Health, Bethesda, Maryland, United States of America; 11 Division of Neurodegenerative Disorders, Hertie Institute for Clinical Brain Research, University of Tubingen, Tubingen, Germany; 12 Pierre and Marie Curie University, Institut National de la Santé et de la Recherche Médicale UMRS 937, Paris, France; 13 CRICM, University Pierre et Marie Curie, Institut National de la Santé et de la Recherche Médicale UMRS 975, CNRS UMR 7225, Hospital Pitié-Salpêtrière, Paris, France; 14 Department of Clinical Genetics, Section of Medical Genomics, VU University Medical Centre, Amsterdam, The Netherlands; 15 Unit of Transgenesis, Laboratory of Neurogenetics, National Institute on Aging, National Institutes of Health, Bethesda, Maryland, United States of America; 16 Institute for Genomics and Multiscale Biology, Mount Sinai School of Medicine, New York, New York, United States of America; Johns Hopkins, United States of America

## Abstract

Association studies have identified several signals at the *LRRK2* locus for Parkinson's disease (PD), Crohn's disease (CD) and leprosy. However, little is known about the molecular mechanisms mediating these effects. To further characterize this locus, we fine-mapped the risk association in 5,802 PD and 5,556 controls using a dense genotyping array (ImmunoChip). Using samples from 134 post-mortem control adult human brains (UK Human Brain Expression Consortium), where up to ten brain regions were available per individual, we studied the regional variation, splicing and regulation of *LRRK2*. We found convincing evidence for a common variant PD association located outside of the *LRRK2* protein coding region (rs117762348, A>G, P = 2.56×10^−8^, case/control MAF 0.083/0.074, odds ratio 0.86 for the minor allele with 95% confidence interval [0.80–0.91]). We show that mRNA expression levels are highest in cortical regions and lowest in cerebellum. We find an exon quantitative trait locus (QTL) in brain samples that localizes to exons 32–33 and investigate the molecular basis of this eQTL using RNA-Seq data in n = 8 brain samples. The genotype underlying this eQTL is in strong linkage disequilibrium with the CD associated non-synonymous SNP rs3761863 (M2397T). We found two additional QTLs in liver and monocyte samples but none of these explained the common variant PD association at rs117762348. Our results characterize the *LRRK2* locus, and highlight the importance and difficulties of fine-mapping and integration of multiple datasets to delineate pathogenic variants and thus develop an understanding of disease mechanisms.

## Introduction

The role of *LRRK2* in human disease was first recognised in 2004 when dominant mutations in the *LRRK2* gene were linked to Parkinson's disease (PD). Rare genetic variants located in the *LRRK2* gene contribute to a significant fraction of familial clustering of the disease [1]. In particular, heterozygous carriers of the non-synonymous change G2019S have an estimated PD lifetime risk close to 50% [2], [3], which directly implicates the gene *LRRK2* as causally implicated in PD aetiology. In addition, recent GWAS results suggest that common variants with a more modest effect on PD risk also exist at this locus [4]. Intriguingly, GWAS have implicated *LRRK2* in the pathogenesis of Crohn's disease (CD) and leprosy [5]–[7]. However, the mechanisms linking PD and the *LRRK2* gene, and more generally the *LRRK2* gene and human disease, remain largely unknown and are the focus of an intense research effort.

The *LRRK2* gene spans 144 kb and is made up of 51 exons. It produces a 2,527 amino acid protein with multiple functional domains, including leucine-rich repeats (LRR), a GTPase domain (Ras of Complex proteins or ROC domain), a domain of unknown function termed the C-terminal of ROC (COR) domain, a kinase domain and a WD40 domain [8]. Coding changes causative for PD are located within the enzymatic core of LRRK2, namely the ROC-COR-Kinase triad of domains, and several of the mutations described in this region disrupt the enzymatic activities of the protein – strongly implicating the enzymatic function of LRRK2 in the pathogenesis of PD [9]–[11]. Existing data demonstrates that *LRRK2* expression is not restricted to the human brain, but is also found in liver, kidney and thymus [12], [13]. A number of studies have been undertaken to investigate the function, expression and cellular localization of *LRRK2* in the human brain [14]–[17]. However, these findings have been based on relatively small numbers of individuals. Consequently, much of the biology of this gene remains unknown. In particular, very little is known about the pattern of *LRRK2* gene expression across the brain, to what extent it is the subject of alternative splicing and, if this is the case, whether splicing is region-specific, and finally how the expression of *LRRK2* is regulated – all information that is likely to be critical for deciphering how *LRRK2* dysfunction results in disease.

A key area of research is the identification of the molecular mechanisms linking variants in the *LRRK2* region with modified risk for PD, CD and leprosy [1], [4], [6], [7], [18]. We hypothesize that some of these effects are mediated by genetic control of the expression and/or splicing or *LRRK2* mRNA. This hypothesis can be tested by combining disease GWAS results with expression datasets and by investigating whether these association signals are compatible with shared causal variants [19]. In addition to suggesting causal mechanisms, this analysis can point to tissue types involved in disease pathogenesis. Therefore, on the basis that PD is driven by pathophysiological processes resulting in the death of neuronal cell populations, there is considerable interest in dissecting the genetic basis of PD susceptibility at the *LRRK2* locus by analysing these results in parallel with expression QTL (eQTL) studies conducted in multiple human tissues.

Here, we use genotype information generated using a custom array (Immunochip) and imputation techniques [20] to provide dense genetic coverage at the *LRRK2* locus from 5,556 controls and 5,802 PD cases of European descent to fine map the PD association at this locus. We compare these data with quantitative, exon-specific *LRRK2* mRNA expression data generated from 10 brain regions originating from 134 neuropathologically-confirmed control individuals also of European descent (1,231 Affymetrix exon arrays). The DNA samples of all 134 individuals were genotyped using the same dense Immunochip array (as well as the Illumina Omni-1M Quad chip). The 10 brain regions analysed include substantia nigra and putamen, brain regions that are relevant to PD pathophysiology. We then combine these results with published GWAS and eQTL findings using imputation techniques [20] in order to obtain insights into the molecular mechanisms of *LRRK2* disease associations.

## Results

### Fine-mapping of the *LRRK2* PD association signals

Using a GWAS of 5,333 cases and 12,019 controls, Nalls et al [4] identified a PD association in the *LRRK2* chromosome region with a common variant (rs1491942, GWAS *P = *3.23×10^−8^, MAF 7%). To fine-map this locus and confirm that this result is independent of the previously described rare variant association at the nsSNP rs34637584/G2019S, we densely genotyped the *LRRK2* region in the GWAS replication set of 5,802 PD cases and 5,556 controls using the ImmunoChip (2.3 typed SNPs per kb on average in the region defined by hg19 chr12:40,351,601-40,830,814, see Methods and [21]). We further increased marker density using 1,000 genomes based imputation (Methods and Table S2).

**Figure 1:**
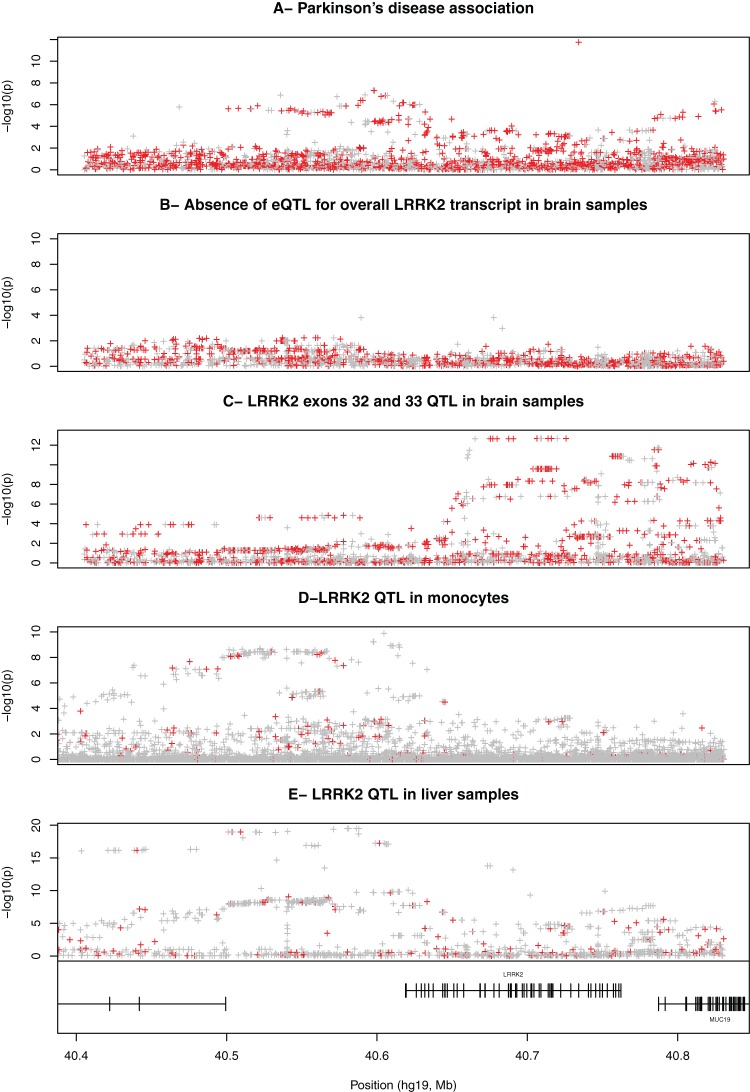
Multiple association signals in the *LRRK2* region chr12:40,351,601-40,830,814. The x-axis shows the physical position (hg19 build) of each variant and the y-axis shows the –log10(p) for association. Typed SNPs are shown in red and imputed SNPs in grey. (**A**) Fine-mapping of the PD association using case-control ImmunoChip genotyping. (**B**) Combined brain expression data across all brain regions and all exons of *LRRK2* (**C**) Exon specific QTL identified in brain samples (combining expression data from ten brain regions). The expression probes showing this signal are located in exons 32 and 33 of *LRRK2.* (**D**) *LRRK2* eQTL identified in 1,490 monocytes samples. (**E**) *LRRK2* eQTL identified in 966 liver samples.

Direct ImmunoChip genotyping in the GWAS replication set confirmed the strong and previously reported association at the rare rs34637584/G2019S non-synonymous SNP (nsSNP, *P* = 1.7×10^−12^, MAF <0.001 in controls, Figure 1A). To investigate the presence of a potential secondary association at this locus, we performed a stepwise conditional regression analysis (Methods). We found that several SNPs located 3′ and 5′ of *LRRK2* remained significant (and with essentially unchanged P-values) after conditioning on the rs34637584/G2019S variant (Figure 1A). The strongest evidence of association in this conditional analysis was found for rs117762348 (imputed A>G SNP with imputation r^2^>0.99, *P* = 2.56×10^−8^, UK control MAF 0.083, UK case MAF 0.074, OR 0.86 with 95% CI: 0.80–0.91 for the minor allele) which is located 5′ of *LRRK2*. rs117762348 is in moderate LD (D' = 1, r^2^ = 0.31, Table S3) with the initial GWAS SNP rs1491942 (combined GWAS and ImmunoChip data for rs117762348 is *P* = 1.55×10^−13^). While other SNPs showed a comparable level of association (Figure 1A), none of them were coding (*P*>10^−4^ for all exonic SNPs, either typed or imputed). After conditioning on both rs117762348 and rs34637584 no other SNP remained significant (*P*>10^−4^). The PD association signal could therefore be summarized using a combination of rs34637584/G2019S and rs117762348.

CD meta-analysis [7] results indicate that at least two independent CD associations (Table 1) exist at the *LRRK2* locus (lead SNPs rs11564258 and rs3761863). The low correlation coefficient (r^2^ = 0.05, Table S3) between these two SNPs is consistent with independent associations. None of the CD SNPs is associated with PD in the immunoChip dataset (Table 1). In contrast, we observed suggestive evidence for PD association in the immunoChip dataset at the leprosy lead SNP rs1491938 (PD *P* = 0.001, OR 1.09, 95% CI: [1.04–1.15], Table 1). However, we found no support for this PD association in the GWAS dataset [4] (*P* = 0.43 for PD at rs1491938).

**Table 1:** Common variant associations in the *LRRK2* region for PD, CD and leprosy.

	Parkinson's disease	Crohn, first signal	Crohn, second signal	Leprosy
**SNP**	rs117762348	rs11564258	rs3761863^a^	rs1491938
**Position**	chr12:40,597,612	chr12:40,792,300	chr12:40,758,652	chr12:40,645,630
**Alleles (major > minor)**	A>G	G>A	C>T	T>C
**MAF**	0.076	0.034	0.32	0.396
**Odds ratio for PD (95% CI) in the immunoChip dataset**	0.85 (0.80–0.91)	0.89 (0.75–1.05)	0.97 (0.91–1.02)	1.09 (1.04–1.15)
**Odds ratio for minor allele (95% CI) for each of the respective disease**	0.85 (0.80–0.91)	1.74 (1.55–1.95)	1.1 (1.05–1.15)	0.86 (0.80–0.92)
**Other SNP in LD (r^2^, Table S3)**	rs1491942 (r^2^ = 0.31, PD GWAS SNP)	-	rs10784486 (r^2^ = 0.7, brain eQTL)	rs10784428 (r^2^ = 0.3, monocyte eQTL)
**P-values for overlap with eQTL datasets (direction of effect for the minor allele)**				
**P-value brain LRRK2 eQTL, exon 32–33 specific**	NS	NS	1.35E-11 (−)	NS
**P-value LRRK2 eQTL liver**	NS	NS	NS	NS
**P-value monocyte LRRK2 eQTL**	9.4E-4 (+)	NS	NS	NS

The CD GWAS results indicate a minimum of two independent associations. For each disease association we list the P-values in the expression datasets. NS: not-significant (*P*>0.001). (a): See Figure S3.

### *LRRK2* mRNA expression levels in different brain regions

The genetic evidence for a regulatory (rather than protein coding) mechanism to explain this common variant association motivated further investigation of *LRRK2* mRNA expression. To assess the pattern of gene expression in human brain, we generated expression data for n = 134 samples without diagnosed neuropathology [22]. Owing to the heterogeneity of the human brain, our brain expression dataset separately quantified mRNA regional levels by Affymetrix GeneChip Human Exon 1.0 ST Arrays in ten brain regions of adult samples (for the majority of the n = 134 samples: 1,231 arrays overall): frontal cortex, occipital cortex (specifically primary visual cortex), temporal cortex, intralobular white matter, thalamus, putamen, substantia nigra, hippocampus, medulla (specifically inferior olivary nucleus) and cerebellum. No significant correlation was found between *LRRK2* mRNA expression level and age/gender. We used these data to assess the extent of brain regional variability in *LRRK2* mRNA expression.

**Figure 2:**
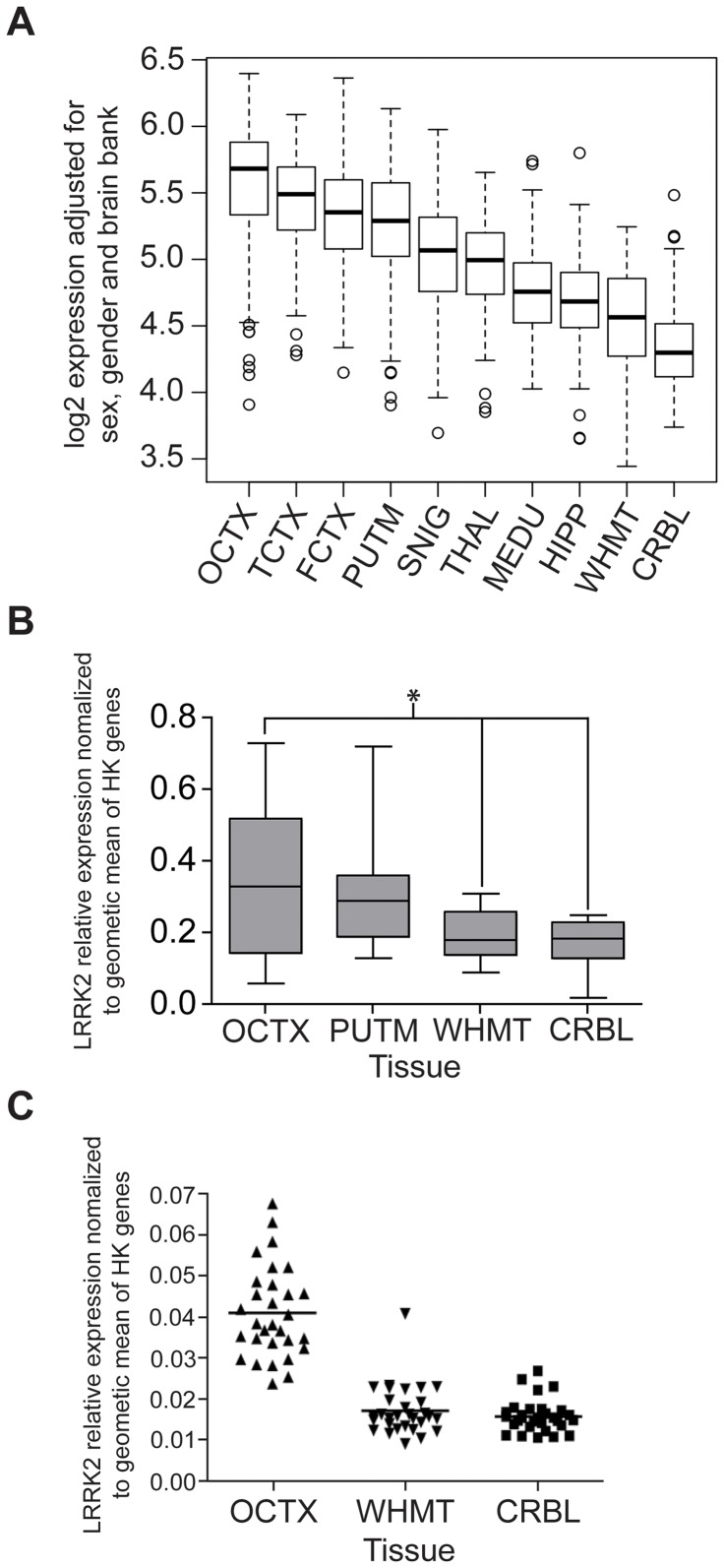
Regional variability in LRRK2 expression. (**A**) Box plot of mRNA expression levels for *LRRK2* in 10 brain regions, based on microarray experiments and plotted on a log2 scale (y axis). Whiskers extend from the box to 1.5 times the inter-quartile range. (**B**) Box plot of mRNA expression levels for *LRRK2* in 4 brain regions, based on QuantiGene experiments. Whiskers extend to the maximum and minimum values. Stars indicate significant differences in expression between brain regions (p-value <0.01, Wilcoxon signed rank testing). (**C**) Dot plot of mRNA expression levels for *LRRK2* in 3 brain regions based on TaqMan Real Time PCR experiments. The expression levels were normalized to the geometric mean of 3 housekeeping genes. The graph shows higher expression in OCTX compared with other regions. Abbreviations: frontal cortex (FCTX), occipital cortex (specifically primary visual cortex, OCTX), temporal cortex (TCTX), intralobular white matter (WHMT), thalamus (THAL), putamen (PUTM), substantia nigra (SNIG), hippocampus (HIPP), medulla (specifically inferior olivary nucleus, MEDU) and cerebellum (CRBL).

Our array results showed evidence of regional variability in mRNA expression patterns. *LRRK2* mRNA levels were two-fold higher in the occipital cortex (analysis of variance *P*<10^−20^), the region expressing the highest *LRRK2* levels, as compared to cerebellum and white matter (Figure 2A). The *LRRK2* expression level in substantia nigra and putamen, the brain regions most implicated in the pathophysiology of PD, were unremarkable (Figure 2A). QuantiGene (QG, a non-PCR method based method, Figure 2B) and real-time qPCR (Figure 2C) were used to confirm the array results and demonstrated similar regional mRNA expression patterns in a subset of brain samples within 4 selected regions. As we have previously documented [22] we found very good agreement between the different gene expression quantification methods.

### Identification of several gene QTLs and exon QTLs for LRRK2

The localization of the secondary PD association signal outside of the *LRRK2* coding region suggests that its effect might be mediated by mRNA expression levels. We therefore used mRNA expression datasets generated by our own group, as well as publically available datasets, to investigate the extent of a genetic control in cis of *LRRK2* mRNA expression levels. In addition to the brain expression dataset that we generated [22], we investigated a total of 15 eQTL expression studies (Table S4). Since each of these studies using probes located in different exons, and these may not capture the expression level of the entire gene, we generally refer to exon eQTL to describe these associations. Only the brain expression datasets covers the majority of *LRRK2*'s exons, whereas the liver (exon 51) and monocyte (exon 50) datasets use single good quality probes.

To perform our exon eQTL analysis we combined our brain expression dataset with liver ([23], n = 970, Methods) and monocytes (Gutenberg Health Study [24], n = 1,490) samples. All expression probes were carefully checked for variants located within them using the 1,000 Genomes dataset and the NHLBI exome sequencing project data (http://evs.gs.washington.edu/EVS/) and excluded if necessary. In brain, and after combining the data across all the exonic probes, we found no significant correlation between overall *LRRK2* gene expression and genotypes (Figure 1B). However, we identified a brain eQTL implicating specifically exons 32 and 33 with rs10784486 (Table 2 and Figure 1C) across all brain regions (Figure S2). We also identified two additional and independent eQTLs (Table 2): one in monocytes (with rs10784428, Figure 1D) and one in liver (with rs11175518, Figure 1E and S1). For the liver and monocyte data, a single good quality exonic probe was present on the array. We therefore expect that these signals capture information about the expression of the overall *LRRK2* gene, in contrast with the brain eQTL that was observed only for exons 32 and 33.

**Table 2:** List of identified *LRRK2* eQTLs in three gene expression datasets: brain (n = 134), liver (n = 970) and monocytes (n = 1,490).

Lead SNP	Position	Alleles	MAF	Probe location	P-value (direction of effect for minor allele)	PD association P-value (estimated OR for minor allele)
**n = 134 brain samples, imputation from Immunochip, Affymetrix exon array**						
rs10784486^a^	chr12:40,677,029	C>A	0.33	exons 32 and 33	2.24E-13 (−)	0.11 (0.95)
**n = 966 liver samples, imputation from Illumina 660W, custom Affymetrix array (exons 5, 19, and 51)**						
rs11175518^b^	chr12:40,580,318	C>T	0.0709	exon 51	4.18E-21 (+)	0.015 (0.88)
**n = 1,372 monocyte samples, probes located in exons 50**						
rs10784428	chr12:40,604,608	C>A	0.44	exon 50	1.3E-10 (−)	0.66 (0.99)

For the P-value computations in brain samples, *LRRK2* expression values are averaged across all 10 brain regions. MAF: minor allele frequency. ^a^: See Figure S2. ^b^: See Figure S1.

We found no significant evidence in the brain samples for an exon eQTL at the liver SNP rs11175518 (*P* = 0.054 in brain) or at the monocyte exon eQTL SNP (*P* = 0.073 in brain for rs10784428), although this may be a consequence of the smaller sample size of the brain expression study.

### Investigation of the RNA mechanism explaining the brain exon 32–33 eQTL

**Figure 3:**
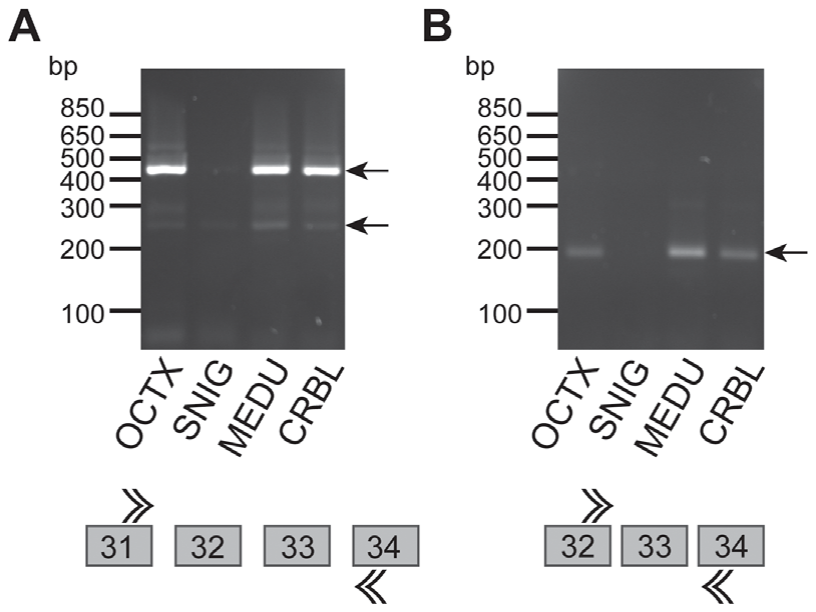
RT-PCR results showing evidence of amplifiable splice forms across exons 32–33 of LRRK2 in selected brain regions, occipital cortex (OCTX), substantia nigra (SNIG), medulla (MEDU) and cerebellum (CRBL). (**A**) RT-PCR results confirming the splicing out of exons 32–33 in SNIG, compared with the other brain regions tested. The expected band size for the isoform with exon 32–33 included is 470 bps, whereas that for the isoform with exon 32 alone spliced out is 270 bps. These results show splicing out of exons 32–33 in substantia nigra and the existence of an isoform with exon 32 alone spliced out in OCTX, MEDU and CRBL. (**B**) RT-PCR results further confirm the splicing out of exon 33 in SNIG. While OCTX, MEDU and CRBL show the expected band size of 195 bps suggesting that exon 32–33 is not spliced out in these regions, SNIG does not.

Given the evidence of an exon specific QTL in exons 32–33 of *LRRK2*, we investigated whether we could identify the RNA mechanism explaining this result. As a first step, we used junction and exon-specific primers (Table S5), and reverse transcriptase PCR (RT-PCR) in 12 randomly selected brain samples and four brain regions to further explore the splicing patterns of exons 32–33. Our data indicate that we can indeed amplify isoforms with spliced out exons 32–33 (Figure 3). These isoforms may however be rare.

In order to better understand the splicing pattern around these exons using a quantitative approach, we generated RNA-Seq data (101 bp reads paired end using the Illumina HiSeq instrument) from total RNA in n = 8 post mortem brain samples (4 brain donors, 2 brain regions: substantia nigra and occipital cortex, Methods). These four samples were selected from the set of n = 134 brain samples with microarray data with the following constraints (in order of priority): male, same ischaemic heart disease as cause of death, similar ages at death and post mortem intervals. The genotypes of these 4 brain donors at the lead brain exon 32–33 eQTL SNP rs11175518 are CC, AC, AA and AA.

We aligned the sequencing reads against the hg19 version of the human reference genome using the STAR aligner [25], which is designed for RNA-Seq alignment, including the incorporation of known exon-exon junctions as well as the discovery of novel ones. We complemented the known junction category by adding the exon junctions 32–34 and 31–34 in the known junction file, to maximize the power to detect these events.

A summary of the number of sequencing reads (mapped and overall) for the RNA-Seq data is provided in Table S6. Table S7 summarizes all junction reads, split into 3 categories: (i) predicted junctions for the canonical *LRRK2* transcript (Ensembl identifier ENST0000029891), (ii) predicted junctions for the non-canonical *LRRK2* transcript (iii) novel junctions. Overall expression of LRRK2 was sufficient to detect all canonical junctions with strong support in all 8 samples (Table S7). The only non-canonical junction observed with strong support (> 10 junction reads) in the region of exons 29–35 involved the skipping of exon 34 (Table S7). Only 3 reads supported the skipping of exon 32 and none skipped exon 33 (Table S7). Based on these data, we conclude that no novel junction event present at high frequency was obviously capable of explaining the exon 32–33 eQTL, leaving this question unsolved. However, we note that the canonical junctions in the exons 29–35 region of *LRRK2* had an overall lower read count than the other *LRRK2* canonical junctions, suggesting that there may be unexplained splicing complexity in this region of *LRRK2*.

### Relevance of genetic control of expression for PD, CD and leprosy

Owing to the multiple exon eQTLs and disease association signals in the *LRRK2* region, we then assessed whether the data are compatible with shared causal variants for eQTLs and disease studies. This analysis identified a mRNA/exon eQTL pair consistent with a shared causal variant. The secondary *LRRK2* CD association (lead SNP rs3761863) was strongly correlated with *LRRK2* mRNA expression of exons 32 and 33 in brain samples (Table 2 and Figure S3). The minor allele T of rs3761863 increases CD risk and is associated with a decrease in *LRRK2* expression (Table 1 and Figure S3). While the low typing density of the latest CD meta-analysis (for the rs3761863/CD exon eQTL) does not enable a formal test of co-localization between disease and eQTL signals [19], these results suggest a plausible mRNA mechanism to mediate this secondary CD association signals. Denser fine-mapping data for CD is required to answer this question.

We also observed a weak association between the PD associated lead SNP rs117762348 and the *LRRK2* eQTL in monocyte cells (*P* = 9.4×10^−4^, Table 1). However, while the lead SNPs for PD (rs117762348) and monocyte exon eQTL (rs10784428) are physically close (separated by 6,996 bp, Figure 1), these two SNPs are not in LD (r^2^ = 0.05, Table S3). Accordingly, the strength of the monocyte exon eQTL association at the PD lead SNP is much weaker than the signal observed at the lead monocyte exon eQTL SNP (*P* = 9×10^−4^ for rs117762348 in Table 1 versus *P* = 1.3×10^−10^ for rs10784428 in Table 2). Hence, the data are not supportive of a shared causal variant for the PD association and the monocyte exon eQTL.

## Discussion

Motivated by the role of *LRRK2* in PD, we fine-mapped this locus in a large case-control collection and found firm support for the presence of a common non-coding variant PD association at this locus (MAF ∼8%). However, several SNPs show comparably high level of PD association and we cannot at this stage make a firm statement about the exact causal variant, but instead highlight a group of SNPs in high LD. The analysis of gene expression datasets highlighted the complexity of the genetic control of *LRRK2,* with at least three independent exon QTLs at this locus. The convincing exon QTL found in brain tissue involves exons 32 and 33 of *LRRK2* but does not co-localize with the PD association. Similarly, the exon QTLs identified in liver and monocyte populations do not co-localize with the PD association.

The most recent meta-analysis results for CD indicate a secondary association for CD, for which the nsSNP rs3761863 is a leading associated variant (*P* = 3.0×10^−6^) [7]. rs3761863/M2397T is also strongly associated with the exon 32–33 brain exon eQTL (lead SNP rs10784486) that we identified (*P* = 1.35×10^−11^, Table 1). The human intestine is heavily innervated and therefore brain tissue may not be irrelevant to CD, but it is also plausible that this exon/expression correlation is shared across other tissue types. Our data suggest a potential mechanism to mediate the CD association but additional CD fine-mapping will be required to test whether the CD and brain exon QTL datasets are fully consistent with a shared variant. We also note that a previous study has associated the same nsSNP rs3761863 with LRRK2 protein stability [26]. These results highlight several factors that complicate the follow-up of associated variants at the *LRRK2* locus in all diseases and in particular PD: the existence of multiple *LRRK2* isoforms, variability in mRNA expression and splicing across brain regions, and localisation of LRRK2 protein to both neuronal and non-neuronal cell types.

On the basis of both exon array and RT-PCR data we predict that alternative isoforms including the splicing of either one or both of exons 32–33 must exist. However, in our brain samples RNA-Seq data, we could not find a strong support for junction reads that support the splicing out of exons of 32 and/or 33. While we cannot fully exclude that unexpected artefacts generated a false positive result for that exon 32–33 specific brain eQTL, an alternative scenario would implicate a more complex set of splicing events, potentially involving more than exons 32–33. Analytical challenges, limited read depth and short length of RNA-Seq reads, may prevent us from characterising this event. The lower number of junctions in the exons 29–35 region of *LRRK2* suggests that this may be the case, but further analysis will be required to confirm this hypothesis.

In summary, this study provides novel insights into *LRRK2* expression, splicing and regulation with a potential link to the etiology of CD. It also highlights the relevance of imputation techniques to provide the dense coverage required to integrate multiple disease association and gene expression studies. The localization of the common variant PD association outside of the coding region of *LRRK2* suggests that it is likely that the effect on disease risk is mediated by control of mRNA expression. However the tissue type where this effect might take place remains an open question. These data, together with our recent analysis of the MAPT locus, illustrate the complexities of defining precisely how risk loci contribute to disease and illustrate that there is as much work required to dissect a locus as to identify it in the first place [27]. We expect that future gene expression studies will increase the quality and quantity of data to maximize the power to dissect the genetic control of *LRRK2* expression in all those diseases in which it is implicated.

## Materials and Methods

### Case control collections

Participating studies were genotyped using the ImmunoChip as part of a collaborative agreement with the ImmunoChip Consortium. Genotyping of the UK cases using the Immunochip was undertaken by the WTCCC2 at the Wellcome Trust Sanger Institute which also genotyped the UK control samples. The constituent studies comprising the IPDGC have been described in detail elsewhere [4]. All samples were of Caucasian origin from the following countries: UK (1,864 cases and 1,271 controls), USA (2,215 cases and 2,807 controls), Dutch (402 cases and 304 controls), German (712 cases and 1,153 controls) and French (363 cases and 267 controls).

### Genotyping

4 ul of 50 ng/ul gDNA and whole genome amplified DNA samples were marked on the Illumina Infinium Omni1-Quad BeadChip (Illumina), which characterizes over 1.1 million SNPs across the genome, according to the manufacturer's instructions. The BeadChips were scanned using an iScan (Illumina) with an AutoLoader (Illumina).

Brain and case control samples were also genotyped using the Immunochip, a custom genotyping array designed for the fine-mapping of auto-immune disorders. The ImmunoChip also contains 2,000 SNPs selected for replication of a Parkinson's disease genome-wide association study performed by the International Parkinson's Disease Genomics Consortium [4], [21]. GenomeStudio v.1.8.X (Illumina Corp.) was used for analysing the data and generating SNP calls. SNP calls were created using the HumanOmni1-Quad_v1-0_B (C) cluster file provided by Illumina as a reference. Standard quality control checks carried out on both dataset include the removal of SNPs that are either labelled as copy number variations, labelled as indels, monomorphic in this data, have less than 95% genotyping rate across samples, deviate from Hardy-Weinberg Equilibrium at p<0.0001, had no genomic position information, had less than two heterozygotes or redundant with an existing SNP. After quality control, we had 815,859 SNPs from the Illumina Omi 1M chip and 137,456 from Immunochip and obtained a total of 905,943 after merging the two dataset and resolving for overlapping SNPs. All manipulation with genotyped data was performed in PLINK version 1.07 [28]. Next, we imputed un-typed SNPs from the autosomes and chromosome X using the MACH [29], [30], minimac software (http://genome.sph.umich.edu/wiki/Minimac) and the 1000 Genomes Project (May 2011 Haplotype release) which is based on 381 individuals of European descent. For this paper, we restricted the imputed data to SNPs with imputation quality (R^2^) greater than 0.5.

### QTL and disease association analysis

We tested the association between each SNP and each expression profile using the R package snpStats (available as part of the suite of packages Bioconductor). We assumed an additive genetic model for each SNP (1 degree-of-freedom trend test) without additional covariates. Unless otherwise specified, computations using brain expression data use the averaged expression values for each sample across the ten regions surveyed.

### Liver and monocyte expression datasets

The liver dataset has been previously described in [23]. It consists of n = 966 liver samples typed using a custom Affymetrix gene expression array, with DNA typed using the Illumina 660W platform. The unique *LRRK2* probe is located in exon 51 of the canonical transcript. The monocyte Gutenberg Health Study dataset has been described in [24]. It consists of n = 1,372 monocyte samples, with expression data measured using the Illumina HT12 BeadChip expression array and DNA was typed using the Affymetrix 6.0 genotyping array. The unique *LRRK2* probe is located in exon 50 of the canonical transcript.

### Human post-mortem brain tissue collection and dissection

Brain and CNS tissue originating from 137 control individuals was collected by the Medical Research Council (MRC) Sudden Death Brain and Tissue Bank, Edinburgh, UK [31], and the Sun Health Research Institute (SHRI) an affiliate of Sun Health Corporation, USA [32].

Samples originating from the MRC Sudden Death Brain and Tissue Bank were removed from whole brains as fresh tissue and anatomical regions of interest were sampled from brain coronal slices at autopsy and immediately flash frozen. In the case of samples originating from the SHRI, whole brains were removed as fresh tissue at autopsy and brain coronal slices were frozen. Anatomical regions of interest were sampled from brain coronal slices on dry ice. In all cases control status was confirmed by a consultant neuropathologist. A detailed description of the samples used in the study, tissue processing, pH determination, dissection, quality controls and rationale for covariate correction and statistical analysis of this data set is provided in Trabzuni et al., 2011 [22]. All samples had fully informed consent for retrieval and were authorized for ethically approved scientific investigation (Research Ethics Committee number 10/H0716/3, The national Hospital for Neurology and Neurosurgery & Institute of Neurology Joint Research Ethics Committee). Brain gene expression data are available within the GEO archive, accession number GSE46706.

### DNA extraction from brain samples

DNA was extracted from human post-mortem brain tissues using Qiagen DNeasy kit (Qiagen, UK). The DNA quality was accessed using ethidium bromide stained agarose gel. The concentration and purity of each DNA sample was assessed using the NanoDrop ND-1000 Spectrophotometer V3.3.0. The concentration of each sample was calculated, together with the ratio of absorbance at 260 nm/280 nm and 260 nm/230 nm.

### RNA isolation, processing, and microarray hybridization

Total RNA was isolated from human post-mortem brain tissues based on the Single-step method of RNA isolation [33] using the miRNeasy 96 kit (Qiagen, UK). The quality of total RNA was evaluated by the 2100 Bioanalyzer (Agilent, UK) and RNA 6000 Nano Kit (Agilent) before processing with the Ambion® WT Expression Kit and Affymetrix GeneChip Whole Transcript Sense Target Labelling Assay, and hybridization to the Affymetrix Exon 1.0 ST Arrays following the manufacturers' protocols. Hybridized arrays were scanned on an Affymetrix GeneChip® Scanner 3000 7G and visually inspected for hybridization artefacts. Further details regarding RNA isolation, quality control and processing are reported in [22].

### Exon array data analysis

All arrays were pre-processed using RMA quantile normalisation with background correction and probe set summarisation with median polish according to the Robust Multi-array Average (RMA) [34] algorithm in Affymetrix Power Tools 1.14.3 (http://www.affymetrix.com/partners_programs/programs/developer/tools/powertools.affx). After re-mapping the Affymetrix probe sets onto human genome build 19 (GRCh37) as documented in the Netaffx annotation file (HuEx-1_0-st-v2 Probeset Annotations, Release 354 31), we restricted analysis to 294,943 probe sets that: i) had gene annotation according, ii) did not target intronic regions and iii) contained at least two 25-mer probes with the following properties: a) unique hybridization to target sequence and b) did not contain SNPs as identified at 1% minor allele frequency from the 1,000 Genomes project (haplotype release May 2011). Since most exons are represented by only one probe set, we used the probe set signal intensity as a synonym of exon expression level, unless explicitly mentioned. Gene-level summary data for 26,684 transcripts was also generated by calculating the 10% trimmed mean of the expression values from the corresponding probe sets. In all types of analysis, the date of array hybridisation, brain bank (SHRI or MRC Sudden Death Brain Bank) and gender were included as co-factors to eliminate confounding effects as investigated in detail in [22].

### Array validation using direct RNA quantification with branched DNA, QuantiGene® 2.0 assay

Cerebellum, occipital cortex, putamen and white matter samples from 12 individuals were analysed using the QG platform for validation of exon array results. We focused on the target gene for validation, Leucine-rich repeat kinase 2 (*LRRK2*). We selected ribosomal protein, large, P0 (RPLP0) and ubiquitin C (UBC) as housekeeping genes to normalize the target genes as they showed relatively low variability in expression levels (i.e. low coefficient of variation) in all brain regions in our dataset. The approach to the selection of reference genes is explained in previous studies [35], [36]. In addition, a recent study confirms the efficiency of using this approach in selecting housekeeping genes to normalize in different tissues [37]. Further details regarding sample processing for this section are reported in Trabzuni et al., 2011 [22].

### Quantitative RT-PCR

Gene expression was quantified by TaqMan real-time PCR technique (Invitrogen, UK) from 30 individuals in cerebellum, occipital cortex and white matter. The *LRRK2* specific assays which cover exon-exon boundary included (Hs00968193), (Hs00411194). Fluorescence was collected using the MxPro system (Agilent, UK). All runs were performed in triplicates and were normalized to a geometric mean of three housekeeping genes (PPIA, RPL0, and UBC). The relative expression values were calculated using the delta delta Ct method (ΔΔCt).

### Semi-quantitative Reverse-Transcriptase RT- PCR

The validation for the array splicing events was done by using QIAGEN Long Range 2step RT-PCR kit (Qiagen, UK). All primers for this analysis were designed using the Primer3 software (fokker.wi.mit.edu/primer3/input.htm), and then they were BLAST searched against UCSC human. In-silico PCR tools. An aliquot of total RNA from 48 samples, originating from four brain regions (occipital cortex, substantia nigra, medulla and cerebellum) and 12 individuals were used as a subset for a further validation. cDNA was synthesis from 1–2 µg of total RNA using gene specific designed primers under the following conditions: Incubation at 42°C for 90 minutes, followed by enzyme activation at 85°C for 5 minutes. 2.5 µl of cDNA was used to perform the Semi-quantitative RT-PCR for the targeted exons under the following conditions: Initial activation at 93°C for 3 min, followed by denaturation at 93°C for 15 sec, annealing 59°C for 50 sec, elongation 68°C for 50 sec for 30–40 cycles. PCR products were run on a 2% agarose gel (Invitrogen, UK) and photographed using UV illumination to visualize GelRed staining. Images were inverted in Adobe Photoshop.

### RNA-Seq data and alignment

cDNA was produced using 100 ng of total RNA and the Ovation RNA-seq system v2 (NuGEN, UK). The concentration of ds-cDNA was determined using the QuBit dsDNA BR assay kit (Invitrogen, UK). Fragmentation of the cDNA was done using a Covaris S220 to a fragment size of 100–900 bp with a median size of 250. DNA libraries were prepared using 1.2 µg cDNA with the TruSeq DNA LT Sample prep kit (illumine, UK). Library concentrations were determined using the KAPA library Quant Kit (KAPAbiosystems) before being pooled into pools of three samples. Each pool was sequenced on a single lane of a Paired End HiSeq v3 Flow cell (Illumina) using a read length of 101 bp for each read. Reads were aligned against the hg19 reference genome using STAR with default parameters. The STAR alignment used the default standard junctions data provided with the software with the addition of junctions 31–34 and 32–34. We then extracted the count data for all junctions (previously known and novel) from the output of STAR.

## Supporting Information

Figure S1 ***LRRK2*** **exon 51 expression stratified by rs11175518 in 966 liver samples.**
(PDF)

Figure S2 ***LRRK2*** **exon 33 expression stratified by rs10784486 in 134 brain samples (all ten brain regions shown as well as combined mean across region).**
(PDF)

Figure S3 ***LRRK2*** **exon 33 expression stratified by rs376186 in 134 brain samples (all ten brain regions shown as well as combined mean across region).**
(PDF)

Table S1 **Full list of IPDGC consortium members.**
(DOCX)

Table S2 **Summary of imputation data and functional role of SNPs in the 479 kb long LRRK2 gene region defined as chr12:40,351,601-40,830,814 (hg19).**
(DOCX)

Table S3 **Summary of the pattern of linkage disequilibrium (correlation coefficient r^2^) between the SNPs mentioned throughout the text.**
(DOCX)

Table S4 **Summary of existing eQTL studies performed in human control tissues and cells with relevance to the detection of exon eQTLs relevant to** ***LRRK2*** **.**
(DOCX)

Table S5 **PCR primers used to characterize the exome 32–33 splicing events.**
(DOCX)

Table S6 **Summary information for the 8 samples (4 individuals and 2 brain regions) with RNA-Seq data.**
(DOCX)

Table S7 **Number of sequencing reads spanning exon-exon junctions in the 8 samples with RNA-Seq data.** Results are organised by: (top) canonical transcript, annotated junctions (middle) non-canonical transcript, annotated junctions (bottom) novel junctions.
(CSV)
