# Supplementary figures and images for: Fine-Mapping, Gene Expression and Splicing Analysis of the Disease Associated LRRK2 Locus
Source: PLoS One. 2013 Aug 13;8(8):e70724. doi: 10.1371/journal.pone.0070724 (PMC3742662; doi:10.1371/journal.pone.0070724)

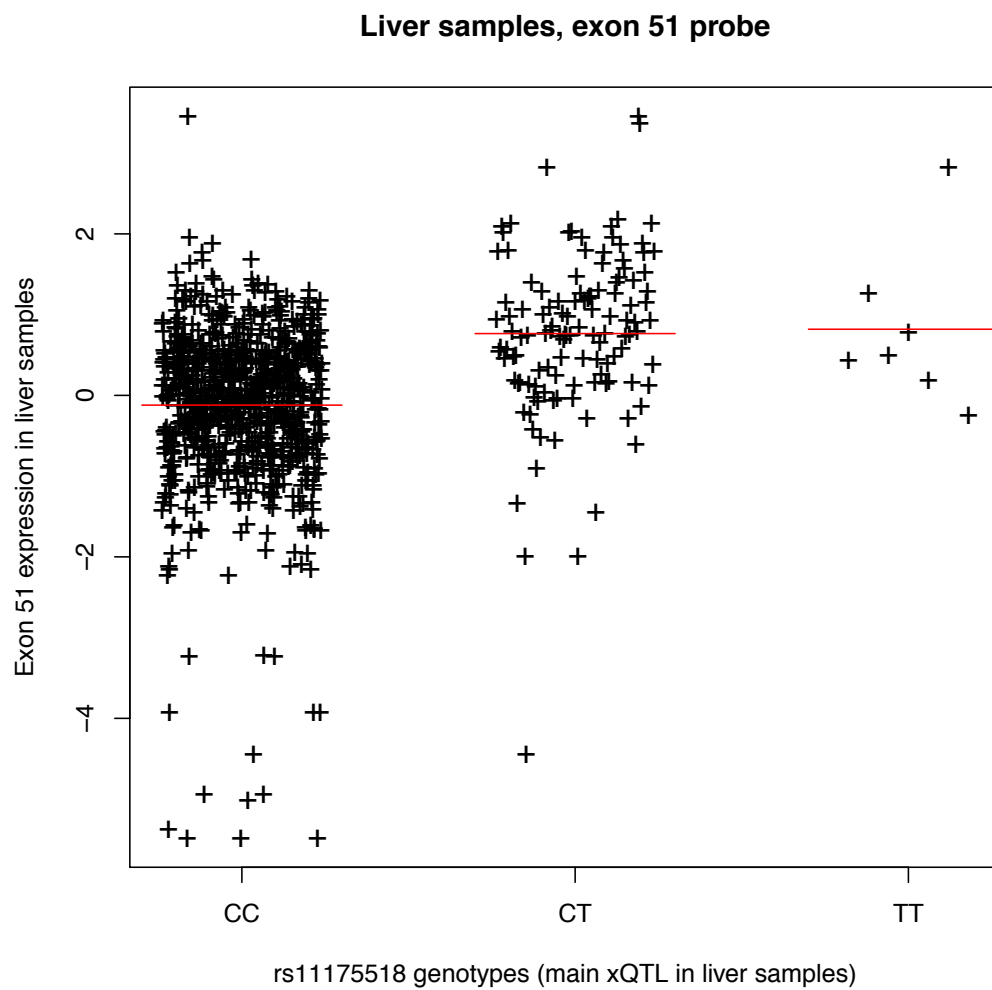

Figure S1: Expression-genotype correlation in 966 liver samples for the SNP rs11175518/exon 51 of *LRKK2*.

Supplement: Figure S1 — LRRK2 exon 51 expression stratified by rs11175518 in 966 liver samples. (PDF) [file pone.0070724.s001.pdf]
