# Supplementary material for: Fine-Mapping, Gene Expression and Splicing Analysis of the Disease Associated LRRK2 Locus
Source: PLoS One. 2013 Aug 13;8(8):e70724. doi: 10.1371/journal.pone.0070724 (PMC3742662; doi:10.1371/journal.pone.0070724)
Supplement: Figure S2 — LRRK2 exon 33 expression stratified by rs10784486 in 134 brain samples (all ten brain regions shown as well as combined mean across region). (PDF) [file pone.0070724.s002.pdf]

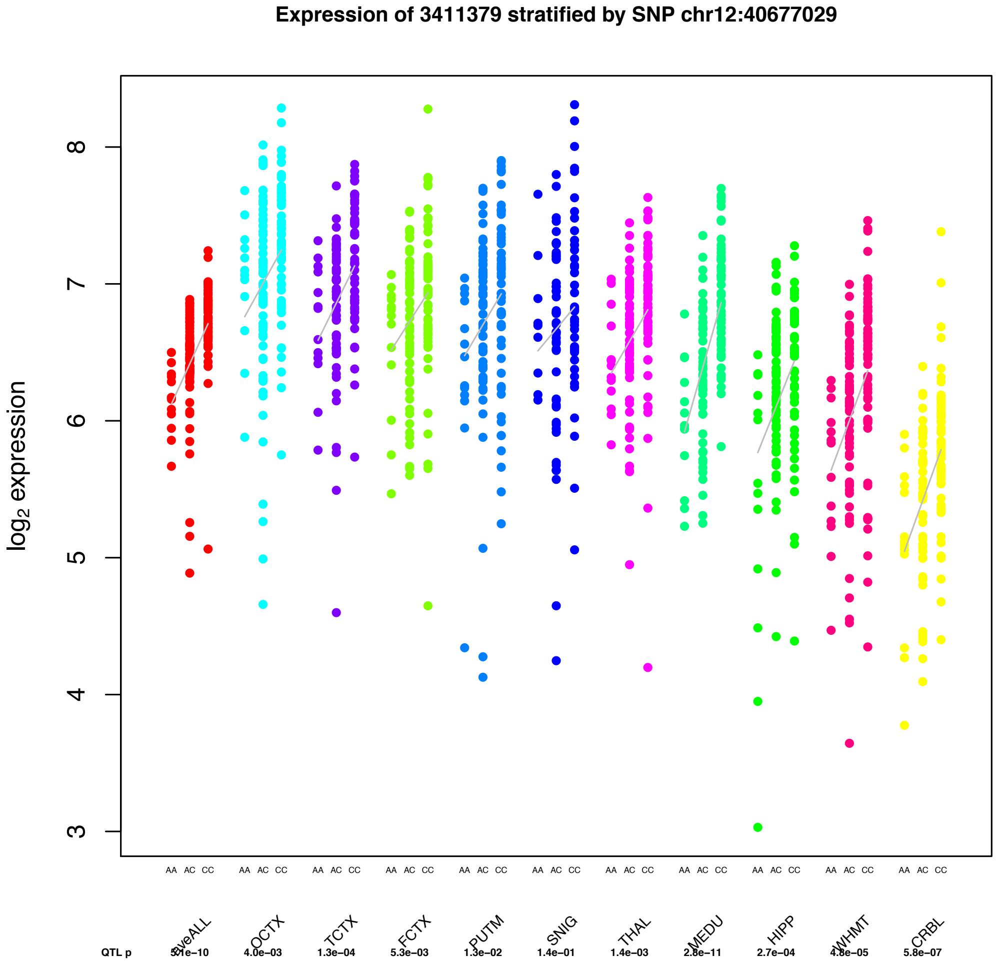

Figure S2: Expression genotype correlation in n = 134 brain samples for exon 33 of *LRRK2* and SNP rs10784486 (main brain xQTL SNP). All ten brain regions are shown.
