# Supplementary material for: Fine-Mapping, Gene Expression and Splicing Analysis of the Disease Associated LRRK2 Locus
Source: PLoS One. 2013 Aug 13;8(8):e70724. doi: 10.1371/journal.pone.0070724 (PMC3742662; doi:10.1371/journal.pone.0070724)
Supplement: Table S2 — Summary of imputation data and functional role of SNPs in the 479 kb long LRRK2 gene region defined as chr12:40,351,601-40,830,814 (hg19). (DOCX) [file pone.0070724.s005.docx]

|  | **Genotyped** | **Imputed (any quality)** | **Imputed (r^2^ > 0.8)** | **Total** |
| --- | --- | --- | --- | --- |
| **Monomorphic** | 255 | 3,285 | - | 3,540 |
| **Polymorphic** | 846 | 1,909 | 1,047 | 2,755 |
| **Total** | 1,101 | 5,194 | 1,047 | 6,295 |
| **Only polymorphic sites** | | | | |
| **Non-synonymous** | 9 | 7 | 3 | 16 |
| **Synonymous** | 9 | 1 | 1 | 10 |
| **Splice change** | 0 | 1 | 0 | 1 |
| **Loss-of-function** | 0 | 0 | 0 | 0 |
| **Intronic** | 443 | 932 | 455 | 1,375 |
| **Intergenic** | 382 | 962 | 588 | 1,344 |
| **UTRs** | 3 | 6 | 4 | 9 |
| **Total** | 846 | 1,909 | 1,047 | 2,755 |

Table S2: Summary of imputation data and functional role of SNPs in the 479 kb long *LRRK2* gene region defined as chr12:40,351,601-40,830,814 (hg19).
