# Supplementary material for: Fine-Mapping, Gene Expression and Splicing Analysis of the Disease Associated LRRK2 Locus
Source: PLoS One. 2013 Aug 13;8(8):e70724. doi: 10.1371/journal.pone.0070724 (PMC3742662; doi:10.1371/journal.pone.0070724)
Supplement: Table S3 — Summary of the pattern of linkage disequilibrium (correlation coefficient r2) between the SNPs mentioned throughout the text. (DOCX) [file pone.0070724.s006.docx]

| **rsid** | **chr12 position**  **(hg19)** | **description** | **rs11175518** | **rs117762348** | **rs10784428** | **rs1491942** | **rs1491938** | **rs10784486** | **rs3761863** | **rs11564258** |
| --- | --- | --- | --- | --- | --- | --- | --- | --- | --- | --- |
| **rs11175518** | 40,580,318 | liver eQTL, 110kb 5’ of *LRRK2* | - | - | - | - | - | - | - | - |
| **rs117762348** | 40,597,612 | best PD SNP, 91 kb 5’ of *LRRK2* | 0.0041 | - | - | - | - | - | - | - |
| **rs10784428** | 40,604,608 | monocyte eQTL, 84 kb 5’ of *LRRK2* | 0.032 | 0.0496 | - | - | - | - | - | - |
| **rs1491942** | 40,620,808 | GWAS SNP, 78 kb 5’ of *LRRK2* | 0.204 | 0.313 | 0.166 | - | - | - | - | - |
| **rs1491938** | 40,645,630 | Leprosy, 43 kb upstream of *LRRK2* | 0.0128 | 0.0444 | 0.301 | 0.002 | - | - | - | - |
| **rs10784486** | 40,677,029 | brain eQTL, exon 32-33, 12 kb upstream of *LRRK2* | 0.0037 | 0.0112 | 0.0187 | 0.0003 | 0.121 | - | - | - |
| **rs3761863** | 40,758,652 | common nsSNP, Crohn signal 2, exon 49 | 0.0034 | 0.004 | 0.0268 | 0.0178 | 0.105 | 0.7 | - | - |
| **rs11564258** | 40,792,300 | Crohn signal 1, 29 kb 3’ of *LRRK2* | 0.0013 | 0.0037 | 0.0098 | 0.08 | 0.0065 | 0.01 | 0.053 | - |

Table S3: Summary of the pattern of linkage disequilibrium (correlation coefficient r^2^) between the SNPs mentioned throughout the text. Linkage disequilibrium was computed in the brain gene expression dataset, including imputation data for SNPs that were not directly genotyped.
