# Supplementary material for: Fine-Mapping, Gene Expression and Splicing Analysis of the Disease Associated LRRK2 Locus
Source: PLoS One. 2013 Aug 13;8(8):e70724. doi: 10.1371/journal.pone.0070724 (PMC3742662; doi:10.1371/journal.pone.0070724)
Supplement: Table S4 — Summary of existing eQTL studies performed in human control tissues and cells with relevance to the detection of exon eQTLs relevant to LRRK2 . (DOCX) [file pone.0070724.s007.docx]

| **Tissue** | **N** | **Population** | **Are QTLs for lrrk2 detected?** | **Pubmed ID** |
| --- | --- | --- | --- | --- |
| **Monocytes** | 1490 | German descent | Yes | [20502693](http://www.ncbi.nlm.nih.gov/pubmed?term=20502693) [[24](#_ENREF_24)] |
| **Liver** | 427 | Caucasian | Yes | [18462017](http://www.ncbi.nlm.nih.gov/pubmed?term=18462017) [[23](#_ENREF_23)] |
| **Adult Brain: Neocortex** | 279 | Caucasian | No | [17982457](http://www.ncbi.nlm.nih.gov/pubmed?term=17982457) [[47](#_ENREF_47)] |
| **Adult Brain: Temporal cortex** | 144 | Caucasian | No | [20485568](http://www.ncbi.nlm.nih.gov/pubmed?term=20485568) [[48](#_ENREF_48)] |
| **Adult Brain: Frontal cortex** | 236 | Caucasian | No | 20485568; [19222302](http://www.ncbi.nlm.nih.gov/pubmed?term=19222302) [[49](#_ENREF_49)] |
| **Developmental Brain Study: Pre-frontal cortex** | 269 | Caucasian & African-American | No | [22031444](http://www.ncbi.nlm.nih.gov/pubmed?term=22031444) [[50](#_ENREF_50)] |
| **Adult Brain: Cerebellum** | 143 | Caucasian | No | 20485568 |
| **Adult Brain: Pons** | 142 | Caucasian | No | 20485568 |
| **Developmental Brain Study: 16 regions** | 57 | Multi-population | No | [22031440](http://www.ncbi.nlm.nih.gov/pubmed?term=22031440) [[51](#_ENREF_51)] |
| **LCLs** | 495 | Multi-population | No | [17873874](http://www.ncbi.nlm.nih.gov/pubmed?term=17873874) [[52](#_ENREF_52)]; [18846210](http://www.ncbi.nlm.nih.gov/pubmed?term=18846210) [[53](#_ENREF_53)]; [20220758](http://www.ncbi.nlm.nih.gov/pubmed?term=20220758) [[54](#_ENREF_54)]; [20220756](http://www.ncbi.nlm.nih.gov/pubmed?term=20220756) [[55](#_ENREF_55)]; 21304890 |
| **T-cells** | 75 | Caucasian | No | [19644074](http://www.ncbi.nlm.nih.gov/pubmed?term=19644074) [[56](#_ENREF_56)] |
| **Fibroblasts** | 75 | Caucasian | No | 19644074 |
| **Skin** | 160 | Caucasian, female twin study | No | [21304890](http://www.ncbi.nlm.nih.gov/pubmed?term=21304890) [[57](#_ENREF_57)] |
| **Blood** | 80 | Caucasian | No | 19222302 |
| **Fat** | 166 | Caucasian, female twin study | No | 21304890 |

**Table S4:** Summary of existing eQTL studies performed in human control tissues and cells with relevance to the detection of exon eQTLs relevant to *LRRK2.*
