# Supplementary material for: Fine-Mapping, Gene Expression and Splicing Analysis of the Disease Associated LRRK2 Locus
Source: PLoS One. 2013 Aug 13;8(8):e70724. doi: 10.1371/journal.pone.0070724 (PMC3742662; doi:10.1371/journal.pone.0070724)
Supplement: Table S5 — PCR primers used to characterize the exome 32–33 splicing events. (DOCX) [file pone.0070724.s008.docx]

| **Exon** | **Primer name** | **Sequence (5′-> 3′)** | **Location** |
| --- | --- | --- | --- |
| 32-33 | LRRK2-Ex32-33 Forward | ACCATCATAAACGAGAGCCTTAATTTC | In exon 31 |
| 32-33 | LRRK2-Ex32-33 Reverse | AGCAATCTGGAATTTTTCTAGGAGCTT | In exon 34 |
| 33 | LRRK2-Exon-33 Forward | CAGCTGCAGTTAGATGAAAATGAGC | In exon 32 |
| 33 | LRRK2-Exon-33 Reverse | TGCCCTTAGGGTGTTTTGGACAACCTT | In exon 34 |

**Table S5:** PCR primers used in this study to characterize the splicing events in the exon 32-33 region.
