# Supplementary material for: Fine-Mapping, Gene Expression and Splicing Analysis of the Disease Associated LRRK2 Locus
Source: PLoS One. 2013 Aug 13;8(8):e70724. doi: 10.1371/journal.pone.0070724 (PMC3742662; doi:10.1371/journal.pone.0070724)
Supplement: Table S6 — Summary information for the 8 samples (4 individuals and 2 brain regions) with RNA-Seq data. (DOCX) [file pone.0070724.s009.docx]

| Sample ID | Number of reads | Uniquely mapped reads | Multi-map reads | Unmapped | Brain region | rs10784486 |
| --- | --- | --- | --- | --- | --- | --- |
| 1 | 59,525,146 | 47,972,124 | 2,522,620 | 15.14% | OCTX | CC |
| 2 | 39,674,488 | 31,919,839 | 1,677,063 | 15.24% | OCTX | AA |
| 3 | 66,601,578 | 54,570,769 | 2,669,418 | 13.97% | OCTX | AC |
| 4 | 66,632,426 | 50,738,769 | 3,598,953 | 18.32% | OCTX | AA |
| 1 | 60,177,454 | 46,600,051 | 2,912,103 | 17.67% | SNIG | CC |
| 2 | 71,112,243 | 54,686,611 | 4,770,894 | 16.36% | SNIG | AA |
| 3 | 72,673,896 | 53,910,413 | 5,087,429 | 18.78% | SNIG | AC |
| 4 | 67,246,545 | 55,740,567 | 2,929,890 | 12.67% | SNIG | AA |

**Table S6:** Summary information for the 8 samples (4 individuals and 2 brain regions) with RNA-Seq data.
